# Days spent with healthcare contact by patients with metastatic castrate resistant prostate cancer in the last year of life

**DOI:** 10.1093/oncolo/oyaf046

**Published:** 2025-05-16

**Authors:** Daniel Sentana-Lledo, Amelia Barnett, David J Einstein, Arjun Gupta, Alicia K Morgans

**Affiliations:** Dana-Farber Cancer Institute, Harvard Medical School, Boston, MA 02215, United States; Beth Israel Deaconess Medical Center, Harvard Medical School, Boston, MA 02215, United States; Beth Israel Deaconess Medical Center, Harvard Medical School, Boston, MA 02215, United States; Masonic Cancer Center at the University of Minnesota, Minneapolis, MN 55455, United States; Dana-Farber Cancer Institute, Harvard Medical School, Boston, MA 02215, United States

**Keywords:** healthcare contact, time toxicity, metastatic prostate cancer, hospice, end-of-life

## Abstract

**Background:**

Days spent with healthcare contact during cancer treatment, or “time toxicity,” may be particularly relevant to patients with end-stage malignancies, including metastatic castrate resistant prostate cancer (mCRPC), considering further cancer-directed therapies near the end-of-life.

**Patients and methods:**

We retrospectively assessed healthcare contact days (ie, days with healthcare contact outside the home) in the last 12 months of 96 patients with mCRPC at an academic cancer center. We compared contact days in the 90-day period between 12 and 9 months prior to death (“first quarter”) and the 90-day period prior to death (“last quarter”) using the Wilcoxon signed rank test, with additional univariate analyses focused on clinicodemographic variables, particularly systemic treatments.

**Results:**

There were higher median [IQR] total contact days in the last vs first quarter (6 [2,17] vs 4 [3,6], *P* < .01), driven by emergency room visits and hospitalizations. Compared to patients off treatment in the first quarter (4 [3,4]), chemotherapy (5 [4,6], *P* = .02) and radiotherapeutics (5 [4,9], *P* = .03) were associated with greater contact days, but not androgen signaling inhibitors (3 [2,5], *P* = .20). However, there were no differences in contact days by treatment in the last quarter. Patients enrolled in hospice experienced similar contact days between the last and first quarters (4 [2,15] vs 4 [3,7], *P* = .22).

**Conclusion:**

Patients with mCRPC experienced increased healthcare contact in the last three months of life resulting from higher inpatient level of care, despite most enrolling in hospice. Future studies can further set expectations on time toxicity in end-stage mCRPC.

**Implication for practice**

This study of time toxicity in patients with mCRPC during their last year of life revealed increasing healthcare contact in the preceding months before death. Patients and providers can use these early findings on expected contact days associated with treatment of mCRPC to frame conversations near the end-of-life.

## Introduction

Prostate cancer is the most prevalent malignancy and the second leading cause of cancer mortality in American men.**^1^** While improvements in treatment have greatly decreased mortality, a proportion of patients eventually progress to metastatic castrate resistant prostate cancer (*mCRPC*). In this incurable stage, treatment focuses on preventing morbidity associated with cancer progression and extending survival while preserving patients’ quality of life.**^2,3^** Throughout their advanced cancer journey, patients and clinicians have conversations weighing the benefit of pursuing additional cancer-directed treatments for disease control with the risk of causing treatment-related toxicity that could increase morbidity and hasten death. At any point, patients can decide to forego cancer treatment and receive supportive care with the aim of maximizing quality of life, usually by enrolling in hospice at home away from the healthcare system.**^4,5^**

While clinicians are generally equipped with the data to discuss the expected efficacy, side effect profile, and prognosis of treatment, there is limited quantitative information available regarding the time commitment associated with specific treatments. Time spent with healthcare contact, or “*time toxicity*,” is a novel outcome measure that quantifies the expected time dedicated to receiving cancer care—including time spent in treatment delivery, monitoring, and managing complications. Any of these may detract from the potential gain in survival time associated with treatment.**^6,7^** Studies of time spent with healthcare contact have mostly focused on patients with advanced malignancies, revealing that healthcare contact appears highest at diagnosis and near the end-of-life.**^8-10^** However, there has not been a dedicated evaluation of time toxicity in the last year of life of patients with cancer, where time becomes increasingly finite and the potential life-prolonging benefits of treatment must be carefully balanced with the risk of increasing time spent in healthcare facilities and reducing time at home. Patients with mCRPC are often elderly and have comorbidities that can limit treatment selection, making time toxicity potentially highly relevant to treatment decision-making for this population. The purpose of this study was to characterize the number and type of days spent with healthcare contact in patients with mCRPC during their last year of life.

## Materials and methods

### Study design

This is a retrospective cohort study evaluating days with healthcare contact in patients with mCRPC receiving medical oncology care at a single academic institution. Eligible patients were treated with systemic antineoplastic therapy in the last 12 months of life and had their date of death documented in the medical record. From an initial 805 potential patients identified using claims-based data - C61 (“Malignant neoplasm of prostate”) together with Z51.1 (“Encounter for antineoplastic chemotherapy and immunotherapy”), 96 met criteria for inclusion in the study. The most frequent reasons for exclusion were the patient being alive, unavailable date of death, and lack of castrate resistance at least 12 months prior to death, in that order. Data collected spanned visits from 1/2015 to 6/2023. Clinical and demographic variables were abstracted from the electronic medical record, including dates and types of physical encounters with the medical system. Distance to the medical center was calculated by determining the average road distance in miles between the patient’s and the medical center’s zip codes. Encounters were categorized as provider clinic visits, outpatient infusions, laboratory studies, imaging, emergency room visits, inpatient hospitalizations, and stays at rehabilitation facilities. We employed the healthcare contact day vs home day model to quantify time toxicity.**^6,7^** In this model, contact days were defined as any day with physical contact with the healthcare system (ie, did not include telehealth visits). A single contact day could contain more than one type of encounter (eg, provider and infusion); in that scenario, contact days were only counted once and assigned to the provider subtype. Days spent in rehabilitation facilities and the date of hospice enrollment were inferred by documentation in the medical record. The de-identified data was recorded in a firewall-protected and encrypted online database.**^11^**

### Endpoints and statistical analysis

The primary outcome of the study was the difference in the days with healthcare contact between the “*first quarter*” (the 90-day period between 12 months to 9 months prior to the date of death) and the “*last quarter*” (the 90-day period immediately prior to date of death). We focused on these two 90-day periods as they were sufficiently apart to detect trends in contact days but remained within an acceptable timeframe of end-stage mCRPC. Based on a prior analysis of time toxicity in patients with metastatic breast cancer during the first 3 months of starting systemic therapy,**^12^** we estimated 10% total contact days (9/90 days) in the first quarter in our study population, while in the last quarter we estimated 30% (27/90 days). Considering a 2-sided alpha of 0.05, power of 0.8, and a common SD of 0.5, we employed paired *t*-test to derive a sample size of 52 patients, of which an additional 15% were extrapolated for a total 60 patients to account for non-parametric data. Ultimately, the study included all 96 eligible patients to provide a stronger signal for exploratory analyses, focused on identifying clinical and demographic predictor variables. Among them, the main secondary endpoint of interest was the difference in contact days associated with the type of systemic treatment, such as androgen signaling inhibitors (ASI) or chemotherapy, during the first and last quarters. Since patients could have switched therapies during the 90-day period in each quarter, for analysis purposes we considered more representative to select the treatment that patients were receiving at the end of that quarter (and if appropriate before hospice enrollment); the results were similar when testing was performed considering treatment patients were receiving at the start of the quarter instead.

Summary statistics were used to highlight the distribution of clinical and demographic variables in the dataset. For the primary outcome, the Wilcoxon signed rank test was employed to detect a difference in contact days between both quarters. Univariate analysis of contact days by clinicodemographic variables was performed using Wilcoxon rank sum and Fisher’s exact test. Data analysis was performed using Stata software, version 17.**^13^**

## Results

Of the 96 patients included in the study, over half were initially diagnosed with Gleason grade group ≥4 prostate cancer, had recurrent metastatic disease, and had bone-predominant metastases. Patients had been living a median of three years with metastatic disease and two years with castrate-resistant prostate cancer. A majority had previously been treated with an ASI, and nearly a third received chemotherapy before the last year of life (**Table 1**).

**Table 1.** Patient demographics and clinical characteristics one year before death.

Characteristics		Patients (*n* = 96)	Characteristics		Patients (*n* = 96)
Mean age (range)		75 (52-97)	Median years with metastatic disease (IQR)		3.3 (2.2, 5.1)
Ancestry (%)	White/Caucasian	69 (71.9)	Lines of metastatic treatment (%)	One	17 (17.7)
	Black/African American	21 (21.9)		Two	29 (30.2)
	Latino/Hispanic	4 (4.1)		Three	28 (29.2)
	Other	2 (1.1)		Four or more	22 (22.9)
Education (%)	High school or less	30 (31.2)	Median years with castrate resistance (IQR)		2.0 (1.2, 3.3)
	Less than college/associates	18 (18.8)	Lines of castrate resistant treatment (%)	One	30 (31.3)
	College or graduate school	48 (50)		Two	35 (36.5)
Insurance (%)	Medicare	76 (79.2)		Three or more	31 (32.2)
	Private	18 (18.7)	Type of prior castrate treatment (%)^*^	Androgen signaling inhibitor	93 (96.9)
	Medicaid/Mass Health	2 (2.1)		*ASI*—abiraterone	65 (67.7)
Median mileage to medical center (IQR)		14.6 (5.3, 29.9)		*ASI*—enzalutamide	59 (61.5)
Gleason grade group (%)	1-2	17 (17.7)		*ASI*—bicalutamide	20 (20.8)
	3	11 (11.5)		*ASI*—apalutamide	2 (2.1)
	4-5	55 (57.3)		*ASI*—darolutamide	2 (2.1)
	Unknown	13 (13.5)		Chemotherapy	29 (30.2)
De novo metastatic (%)	No	64 (66.7)		*Chemotherapy*—docetaxel	29 (30.2)
	Yes	32 (33.3)		*Chemotherapy*—cabazitaxel	3 (3.1)
Site of metastasis (%)^*^	Bone	75 (78.1)		Radium 223	18 (18.8)
	Lymph nodes	36 (37.5)		PARP inhibitor	4 (4.2)
	Visceral	25 (26)		Pembrolizumab	3 (3.1)
				Clinical trial	14 (14.6)

^*^Patients could have more than one type of metastatic site or received more than one form of castrate treatment.

In terms of the primary objective, there were higher median [IQR] total contact days in the last quarter compared to the first quarter (6 [2,17] vs 4 [3,6], *P* < .01). Examining the type of contact days driving this difference, more contact days were spent receiving inpatient level of care in the last quarter than in the first quarter, both in the emergency room (0 [0,1] vs 0 [0,0], *P* < .01) and in the hospitalized setting (0 [0,10] vs 0 [0,0], *P* < .01). There was no difference between total contact days in the last vs first quarter dedicated to provider visits (3 [1,5] vs 3 [2,4], *P* = .15) or infusions (0 [0,1] vs 0 [0,0], *P* = .11). The observed differences were also observed when mean days (rather than median days) were compared (**Table 2**).

**Table 2.** Comparison of median [IQR]/mean (SD) contact days between first and last quarters.

	First quarter (*n* = 96)	Last quarter (*n* = 96)	(*P*-<0.05)^*^
Total contact days	4 [3,6]/5.1 (4.25)	6 [2,17]/10.2 (10.5)	**<.01**
Provider	3 [2,4]/3.7 (2.5)	3 [1,5]/3.3 (2.4)	.15
Infusion	0 [0,1]/0.5 (0.9)	0 [0,0]/0.3 (0.8)	.11
Laboratory	0 [0,0]/0.2 (0.1)	0 [0,0]/0 (0.4)	.57
Imaging	0 [0,1]/0.5 (0.8)	0 [0,0]/0.3 (0.7)	**.01**
ER	0 [0,0]/0.1 (0.5)	0 [0,1]/0.7 (1.0)	**<.01**
Hospitalization	0 [0,0]/0.4 (2.2)	0 [0,10]/5.1 (7.6)	**<.01**
Rehabilitation facility	0 [0,0]/0 (0)	0 [0,0]/0.5 (2.9)	.12

^*^Reflects comparison between median contact days; significance was maintained with mean days.

Any bolded *P*-values in any of the tables means that this was a statistically significant association (*P* < 0.05).

**Figure 1.**
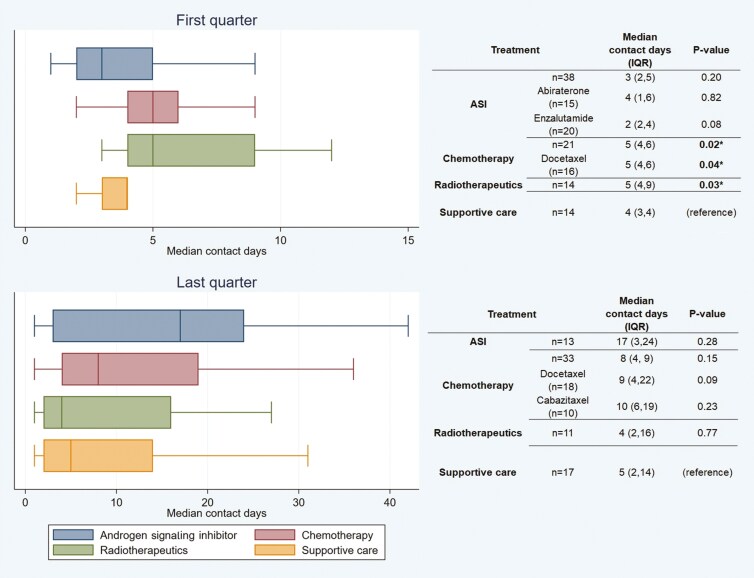
Contact days in first and last quarter by type of treatment.

The most common treatments patients received in the first quarter were ASIs (40%) and chemotherapy (22%); most treated patients in the last quarter were receiving chemotherapy (34%) or ASI (14%). There were a similar number of patients off treatment but not yet enrolled in hospice in both the first (15%, 14/96) and last (18%, 17/96) quarters (**Table 3**). In the first quarter, compared to patients off treatment, patients on chemotherapy had more median total contact days (5 [4,6] vs 4 [3,4], *P* = .02), as did those on radiotherapeutics (5 [4,9] vs 4 [3,4] *P* = .03), but not patients on ASI (3 [2, 5] vs 4 [3,4], *P* = .20). However, in the last quarter, there were no significant differences in contact days between ASI (17 [3,24] vs 5 [2,14], *P* = .28), chemotherapy (8 [4,19] vs 5 [2,14], *P* = .15), or radiotherapeutics (4 [2,16] vs 5 [2,14], *P* = .77) compared to patients off treatment (**Figure 1**). Comparing median total contact days by type of treatment received in the last and first quarters, there were more contact days in patients receiving ASI in the last compared to the first quarter (17 [3,24] vs 3 [2,5], *P* < .01) and there was a non-significant trend for patients on chemotherapy as well (8 [4,19] vs 5 [4,6], *P* = .10); conversely patients on radiotherapeutics (4 [2,16] vs 5 [4,9], *P* = .32) and off treatment (5 [2,14] vs 4 [3,4], *P* = .44) had similar contact days in the last and first quarters.

**Table 3.** Treatment patterns for patients in the first and last quarters.

Treatment received during first quarter		First clinic visit *n* = 96 (%)	Last clinic visit *n* = 96 (%)
ASI	All	51 (53.1)	38 (39.5)
	Enzalutamide	26 (27.1)	20 (20.8)
	Abiraterone	20 (20.8)	15 (15.6)
	Darolutamide	2 (2.1)	2 (2.1)
	Bicalutamide	2 (2.1)	1 (1.0)
	Apalutamide	1 (1.0)	0
Chemotherapy	All	15 (15.6)	21 (21.9)
	Docetaxel	12 (12.5)	16 (16.7)
	Cabazitaxel	3 (3.1)	5 (5.2)
Radium-223		11 (11.4)	14 (14.6)
PARP inhibitor		4 (4.2)	3 (3.1)
Pembrolizumab		2 (2.1)	2 (2.1)
Clinical trial		4 (4.2)	4 (4.2)
Supportive care/Off treatment		9 (9.4)	14 (14.6)
*Switched treatment during first quarter*		*44 (45.8)*	

Treatment received during last quarter		First clinic visit *n* = 96 (%)	Last clinic visit *n* = 96 (%)
ASI	All	15 (15.7)	13 (13.6)
	Abiraterone	9 (9.4)	6 (6.3)
	Enzalutamide	4 (4.2)	5 (5.2)
	Darolutamide	2 (2.1)	2 (2.1)
Chemotherapy	All	25 (26.1)	33 (34.4)
	Docetaxel	13 (13.6)	18 (18.8)
	Cabazitaxel	9 (9.4)	10 (10.4)
	Mitoxantrone	2 (2.1)	4 (4.2)
	Carboplatin	0	1 (1.0)
Radiotherapeutics	All	11 (11.4)	11 (11.4)
	Radium-223	10 (10.4)	10 (10.4)
	Lutetium-177	1 (1.0)	1 (1.0)
PARP inhibitor		1 (1.0)	2 (2.1)
Pembrolizumab		1 (1.0)	1 (1.0)
Clinical trial		4 (4.2)	4 (4.2)
Supportive care/off treatment		24 (25.0)	17 (17.7)
Hospice^*^		15 (15.6)	15 (15.6)
*Switched treatment during last quarter*		*17 (17.7)*	

^*^Patients listed under hospice were enrolled prior to first or last clinic visit respectively; most of the patients who enrolled in hospice enrolled after the last clinic visit.

In the first quarter, patients with college education had more contact days than the median (5 [3,7] vs 4 [3,6], *P* = .02), while those who had lived ≥5 years with metastatic disease had fewer contact days (3 [2,4] vs 4 [3,6], *P* = .02). In the last quarter, there were more contact days compared to the median in patients with Gleason grade group ≥4 (8 [3,19] vs 6 [2,17], *P* = .01) and patients living with metastatic disease for ≤2 years (13 [5,24] vs 6 [2,17], *P* < .01), while those with Gleason grade group ≤2 (3 [2,4] vs 6 [2,17], *P* = .03) and patients living further away from the medical center (2 [1.5] vs 6 [2,17], *P* = .01) had fewer last quarter contact days compared to the median. Analyzing which demographic covariates were associated with a difference in contact days between the last and first quarter, patients who had at most completed high school (9 [2,19] vs 4 [3,5], *P* = .01), and those with supplemental private insurance (14 [2,23] vs 4 [2,6], *P* = .03) had increased healthcare contact in the last quarter. There was no difference by self-reported ancestry. Additionally, patients with Gleason grade group≥4 disease (8 [3,19] vs 4 [2,6], *P* < .01), recurrent prostate cancer (7 [2,19] vs 4 [3,6], *P* < .01), bone metastases (6 [2,18] vs 4 [3,6], *P* = .01), and ≤2 years living with metastatic disease (13 [5,24] vs 5 [2,6], *P* < .01) also had increased contact days in the last compared to the first quarter. There was no single demographic or clinical variable that was independently associated with median total contact days in both the first and last quarters (**Table 4**). Finally, in analyzing patients in the top and bottom quartiles of contact days in each quarter, there were no new evident covariates to explain the differences in healthcare contact in those patient cohorts (*data not shown*).

**Table 4.** Univariate analysis of demographic and clinical variables and its association with median total contact days in first and last quarters.

Variable		Median contact days first quarter (IQR)	(*P*-<0.05)	Median contact days last quarter (IQR)	(*P*-<0.05)	(*P*-<0.05) first vs last quarter
Median contact days (*n* = 96)		4 (3, 6)	*Ref*	6 (2, 17)	*Ref*	<.01
Age	≤ 69 (25th percentile) (*n* = 27)	5 (3, 6)	.46	7 (2, 17)	.72	.04
	≥ 80 (75th percentile) (*n* = 27)	3 (2, 5)	.11	6 (2, 24)	.54	.03
Ancestry	White/Caucasian (*n* = 69)	4 (3, 6)	.39	5 (2, 14)	.31	.05
	Black/African American (*n* = 21)	4 (3, 6)	.49	9 (2, 19)	.37	.03
Education	High school or less (*n* = 30)	4 (3, 5)	.25	9 (2, 19)	.25	.01
	Less than college/associates (*n* = 18)	4 (2, 5)	.11	4 (3, 19)	.53	.07
	College or graduate school (*n* = 48)	5 (3, 7)	.02	5 (1, 13)	.12	.49
Insurance	Medicare (*n* = 76)	4 (3, 6)	.51	5 (2, 14)	.09	.11
	Private (*n* = 18)	4 (2, 6)	.93	14 (2, 23)	.24	.03
Median mileage to medical center	≤ 5.3 (25th percentile) (*n* = 26)	5 (2,7)	.56	11 (3,19)	.31	.02
	≥ 29.9 (75th percentile) (*n* = 25)	4 (3,6)	.60	2 (1,5)	.01	.22
Gleason grade group	1-2 (*n* = 17)	5 (4, 6)	.14	3 (2, 4)	.03	.06
	3 (*n* = 11)	4 (3,7)	.91	5 (1, 14)	.56	.59
	4-5 (*n* = 55)	4 (2, 6)	.76	8 (3, 19)	.01	<.01
	Unknown (*n* = 13)	3 (2, 5)	.20	4 (1, 9)	.50	.50
De novo metastatic	No (*n* = 64)	4 (3, 6)	.44	7 (2, 19)	.27	<.01
	Yes (*n* = 32)	5 (3, 7)		4 (2, 15)		.33
Site of metastasis	Bone (*n* = 75)	4 (3, 6)	.12	6 (2, 18)	.43	.01
	Lymph nodes (*n* = 36)	4 (3, 6)	.77	4 (2, 17)	.40	.20
	Visceral (*n* = 25)	4 (3, 5)	.98	7 (1, 17)	.97	.06
Median years with metastatic disease	≤ 2.2 (25th percentile) (*n* = 25)	5 (2, 6)	.79	13 (5, 24)	<.01	<.01
	≥ 5.1 (75th percentile) (*n* = 25)	3 (2, 4)	.02	5 (1, 14)	.62	.09
Lines of metastatic treatment	One (*n* = 17)	4 (2, 5)	.47	6 (3, 24)	0.32	.05
	Two (*n* = 29)	3 (2, 7)	.42	11 (4, 19)	.06	.02
	Three (*n* = 28)	4 (3, 7)	.57	3 (1, 11)	.07	.92
	Four or more (*n* = 22)	4 (4, 6)	.35	5 (2, 9)	.33	.60
Median years with castrate disease	≤ 1.2 (25th percentile) (*n* = 24)	5 (4, 7)	.35	10 (4, 24)	.05	.02
	≥ 3.3 (75th percentile) (*n* = 25)	3 (3, 6)	.17	3 (3, 6)	.77	.08
Lines of castrate treatment	One (*n* = 30)	4 (2, 6)	.09	8 (3, 22)	.11	<.01
	Two (*n* = 35)	4 (2, 9)	.54	6 (1, 17)	.85	.16
	Three or more (*n* = 31)	4 (4, 6)	.30	3 (2, 9)	.07	.96
Type of prior castrate treatment	ASI (*n* = 93)	4 (3, 6)	.77	6 (2, 17)	.51	<.01
	ASI—*abiraterone* (*n* = 65)	4 (3, 6)	.44	5 (2, 16)	.41	.10
	ASI—*enzalutamide* (*n* = 59)	4 (3, 6)	.88	5 (2, 15)	.36	.12
	ASI—*bicalutamide* (*n* = 20)	4 (4, 5)	.86	6 (2, 22)	.77	.05
	Docetaxel (*n* = 29)	4 (4, 6)	.22	5 (2, 11)	.24	.75
	Radium 223 (*n* = 19)	5 (3, 6)	.33	5 (2, 9)	.80	.59
	Clinical trial (*n* = 14)	6 (4, 7)	.10	8 (2, 13)	.95	.29

Subgroups with *n* < 10 were excluded from the univariate analysis. Wilcoxon rank-sum test was used for unequal-sized groups, while Wilcoxon signed rank test was used for same-sized groups. Two-sided *P*-value unless specified.

**Figure 2.**
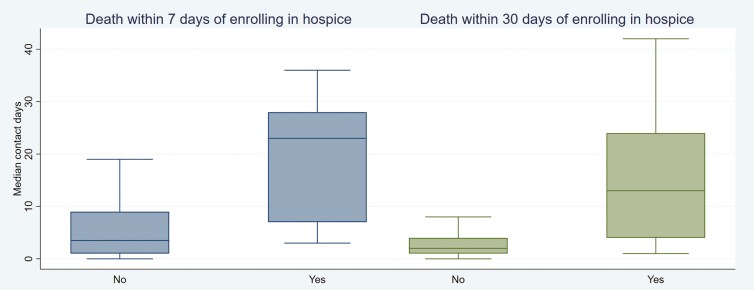
Contact days in the last quarter by timing of hospice enrollment.

Most patients (*n* = 73) enrolled in hospice by the time of death, with patients spending close to a month enrolled (median days 27 [13, 76]). Patients who did not enroll in hospice by time of death (*n* = 23) had more contact days in the last vs first quarter (11 [3,19] vs 3 [2,4], *P* < .01), while patients who did enroll on hospice before death had similar contact days across both quarters (4 [2,15] vs 4 [3,7], *P* = .22). Still, compared to the median there were significantly more contact days in the last quarter for those who enrolled in hospice 7 days (23 [7,28] vs 6 [2,17], *P* < .01) and 30 days (13 [4,24] vs 6 [2,17], *P* < .01) before death (**Figure 2**). Before enrolling in hospice, patients that had received 2 or less lines of metastatic treatment had significantly higher contact days in the last vs first quarter (15 [5, 27] vs 3 [2,4], *P* = .01), while those that had trialed 5 or more lines of treatment had similar contact days between both quarters (3 [2,7] vs 5 [4,6], *P* = .35). Specifically, there was no difference in last vs first quarter contact days if patients had received prior ASI, chemotherapy, and radiotherapeutics (4 [2,9] vs 5 [4,7], *P* = .48), enrolled in a clinical trial (5 [3,9] vs 6 [5,7], *P* = .82), or not received chemotherapy (3 [1,14] vs 2 [2,6], *P* = .41) before hospice enrollment. Of the patients not enrolled in hospice (*n* = 23), close to half (44%) were still receiving chemotherapy and a quarter (26%) were on ASI at the time of death. Of all patients, 6 patients (6.3%) died in the hospital (**Table 5**).

**Table 5.** End-of-life healthcare utilization and univariate analysis.

Variable		Median contact days first quarter (IQR)	(*P*-<0.05)	Median contact days last quarter (IQR)	(*P*-<0.05)	(*P*-<0.05) of first vs last quarter
Median contact days (*n* = 96)		4 (3, 6)	*Ref*	6 (2, 17)	*Ref*	<.01
Enrolled in hospice	Yes (*n* = 73)	4 (3, 7)	<.01	4 (2, 15)	.12	.22
	No (*n* = 23)	3 (2, 4)		11 (3, 19)		<.01
Days since last treatment	≤ 43 (25th percentile) (*n* = 24)	4 (3, 6)	.83	12 (6, 21)	.01	<.01
	≥ 139 (75th percentile) (*n* = 25)	4 (4, 7)	.10	1 (1,4)	<.01	.09
Days since last clinic visit	≤ 26 (25th percentile) (*n* = 27)	4 (3, 6)	.72	13 (5, 23)	<.01	<.01
	≥ 82 (75th percentile) (*n* = 25)	3 (2, 5)	.25	1 (1, 12)	<.01	.97
Days enrolled in hospice	≤ 13 (25th percentile) (*n* = 20)	4 (3, 6)	.93	12 (5, 25)	.01	<.01
	≥ 76 (75th percentile) (*n* = 42)	4 (3, 6)	.65	2 (1, 12)	<.01	.83
Timing of death after enrolling in hospice	7 days (*n* = 11)	4 (2, 6)	.09	23 (7, 28)	<.01	<.01
	30 days (*n* = 39)	4 (3, 6)	.06	13 (4, 24)	<.01	<.01
Lines of metastatic therapy before hospice (n = 73)	Two or less (*n* = 12)	3 (2, 4)	.03	15 (5, 27)	.10	<.01
	Three (*n* = 17)	4 (3, 9)	.65	2 (1, 4)	.02	.26
	Four (*n* = 22)	6 (4, 9)	.02	11 (3, 18)	.34	.15
	Five (*n* = 22)	5 (4, 6)	.03	3 (2, 7)	.09	.35
Lines of castrate therapy before hospice (n = 73)	Two or less (*n* = 12)	3 (2, 4)	.05	11 (4, 25)	.27	.01
	Three (*n* = 19)	4 (3, 9)	.35	3 (1, 14)	.15	.61
	Four (*n* = 22)	5 (4, 7)	.13	6 (3, 18)	.63	.16
	Five (*n* = 20)	5 (4, 7)	.02	3 (2, 8)	.10	.28
Prior ASI, chemotherapy, and radiotherapeutics before hospice (*n* = 33)		5 (4, 7)	<.01	4 (2, 9)	.17	.48
Prior clinical trial before hospice (*n* = 17)		6 (5, 7)	<.01	5 (3, 9)	.60	.82
No prior chemotherapy before hospice (*n* = 11)		2 (2, 6)	.04	3 (1, 14)	.34	.41

Subgroups with *n* < 10 were excluded from the univariate analysis. Two sided *P*-value unless specified.

## Discussion

This study identified trends associated with the time spent with healthcare contact by patients with mCRPC in their last year of life. The development of time toxicity as an outcome measure is relatively new, and to the best of our knowledge, this is the first study to examine the contact days experienced by patients with prostate cancer. Our results show that patients spent an increased number of days with healthcare contact in their last three months of life compared to the beginning of their last year of life despite a majority of patients being enrolled in hospice. While the difference in total median days between both quarters may appear relatively small, there was a noticeably wider range of contact days in the last quarter, reflecting the diverging trajectories of patients with early hospice enrollment and those with high inpatient care needs near death.

The main driver of increased healthcare utilization in the last quarter appeared to be higher contact with inpatient care through hospitalizations. This may not be surprising as patients with mCRPC experience more morbidity related to their disease and treatments closer to the end-of-life, often requiring hospitalization for correcting life-threatening complications.**^14,15^** An analysis of Austrian patients with mCRPC identified high levels of inpatient level of care in the last year of life, with a median of 39 days spent hospitalized and up to 2/3 of patients dying in the hospital.**^16^** Furthermore, a small study in the United Kingdom identified 82% patients with mCRPC requiring inpatient admission in the last year of life, with a mean 10.5 days spent inpatient.**^17^** In contrast to these studies, most of our cohort was enrolled in hospice by the time of death and avoided dying in the hospital, although this could be ascribed to distinct care models. Older studies in the United States described decreasing inpatient level of care requirements in patients dying from prostate cancer over the years, as well as rising use of hospice services**^18,19^**; notably these analyses covered the early 2000s when the mCRPC treatment landscape was different. Nonetheless, studies have consistently shown that patients dying from cancer value avoiding hospitalization towards the end-of-life and prefer to die at home.**^20,21^** While clinicians do not consistently predict patients’ short-term survival,**^22-24^** time toxicity data for the mCRPC population can be utilized in shared decision-making when patients are facing later lines of cancer-directed therapy or leaning towards proceeding with supportive management in a hospice setting at home. In particular, our findings suggest that patients receiving three or more lines of treatment at mCRPC would particularly benefit from this discussion.

Compared to studies in other cancer populations, we found a relatively low proportion of contact days in both 90-day periods for patients with mCRPC. For instance, patients with advanced non-small cell lung cancer in a Canadian cohort who died from their disease had 36 median contact days with the healthcare system, reflecting 33% of total survival days.**^9^** Furthermore, patients with advanced gastrointestinal malignancies in the VA system who similarly died from their cancer had 44 median healthcare contact days, representing 35% of survival time.**^10^** Lastly, another single institution cohort of patients with advanced gastrointestinal cancer spent 25% of their time alive with healthcare contact.**^25^** In contrast, in our cohort, the median contact days in the last quarter was under 10% of total days, which matched the pre-study assumption for *first* quarter contact days. There are several reasons that could explain these differences. To begin with, the studies in other advanced malignancies focused on a longer time period (usually a year) than the two 90-day periods studied in this population, although the percentage of days in our study was still much lower.**^9,10,25^** Secondly, even at end-stage mCRPC, many patients have lower morbidity associated with their malignancy than is experienced by patients with other cancers for a variety of reasons, including rate of tumor growth, availability of effective therapies, and metastatic site-related complications.**^26^** While the driver of higher contact days in the last quarter were emergency department visits and hospitalizations, only 11 of 96 patients in this study required these unplanned care visits. Moreover, most patients in our cohort had enrolled in hospice before death, which likely decreased healthcare contact from what it could have been without hospice support. Prior studies have not explicitly reported the effect of hospice enrollment in contact days. However, one study comparing contact days with treatment vs supportive care in patients with metastatic colorectal cancer did reveal lower contact days in those receiving supportive care,**^8^** and a different analysis of time spent receiving antineoplastic treatment compared to best supportive care across malignancies revealed up to a waking day per month was dedicated to receiving treatment.**^27^** Ultimately, care coordination in our cohort appeared high, particularly in the first quarter, as most patients had limited additional outpatient visits outside of provider visits for labwork or nursing administered treatments. As such, the healthcare delivery patterns of an urban academic medical oncology center may not reflect healthcare contact in other settings. Future studies evaluating the trajectory of patients with mCRPC for longer than these 2 discrete 90-day periods and in more diverse cohorts (ie, geographic location, cancer practice) could offer more precise estimates of contact days.

While not the primary objective, a key secondary aim of the study was to determine whether treatment patterns influenced days with healthcare contact across both the first and last quarters of the last year of life. Indeed, an essential motivation for studying time toxicity is to determine the expected healthcare contact associated with a particular treatment compared to another option or to no treatment at all to optimally counsel patients about what they can expect life to look like along different pathways.**^6,8^** While there were more contact days in the first quarter with infusional regimens like chemotherapy or radiotherapeutics compared to patients receiving supportive care or those receiving an oral ASI, this was not observed in the last quarter. There are several potential explanations for this observed disparity. Perhaps the biggest factor was that hospice enrollment was much higher in the last quarter and the number of patients receiving cancer-directed treatment for the whole quarter was noticeably smaller. Even though there was no statistical difference across treatments in the last quarter, the median total contact days in the quarter trended towards higher healthcare contact in patients on ASI and chemotherapy, likely reflecting insufficient power for this subgroup analysis. The presence of infusional treatments like chemotherapy as a predictor of higher time toxicity has been previously demonstrated,**^12^** as they require administration in a healthcare setting as well as periodic laboratory monitoring. Nonetheless, it is interesting that patients on ASI in the last quarter appeared to have higher contact days while those in the first quarter did not. This paradoxical trend could reflect that patients on ASI as the last line of mCRPC treatment were probably frail, not deemed candidates for systemic treatments with higher risk of toxicity, and thus compromised for an oral, home-based option.**^28^** Overall, the information learned on the healthcare contact with each treatment class in mCRPC should be considered hypothesis-generating until replicated in larger, appropriately powered cohorts.

On exploratory univariate analysis, there were no clear clinical or demographic variables associated with increased healthcare contact in both the first and last quarters, likely reflecting the multifactorial nature of time toxicity. Within each quarter, several covariates appeared related to differences in healthcare contact compared to median total contact days. Notably, years living with metastatic disease showed an interesting pattern in each quarter—patients above the 75th percentile (≥5.1 years) had less healthcare contact in the first quarter, while those in the 25th percentile (≤2.2 years) had significantly higher healthcare contact in the last quarter. Separately, patients with Gleason grade group ≤2 had less contact days in the last quarter than those with Gleason grade group ≥4 disease; however no significant differences in contact days were observed with de novo diagnosis or site of metastasis, both known clinical risk factors associated with poorer prognosis in metastatic prostate cancer.**^29,30^** Finally, comparing the difference in median contact days across quarters, there was no difference by self-reported ancestry, however, patients who had not attended higher education had significantly more contact days in the last vs first quarter, as did patients with private insurance, which is consistent with prior literature on factors associated with increased healthcare expenditures by cancer patients at the end-of-life.**^31^**

Regarding end-of-life healthcare utilization patterns, our findings suggest that hospice was protective against increased contact days in the last quarter of life. Specifically, earlier enrollment was associated with a decrease in healthcare contact. Prior studies in cancer populations identified that 30% of patients enrolled within 7 days prior to death, and that median hospice enrollment was 14.5 days.**^32,33^** Recent nationwide data collected from CMS/Medicare revealed that the national average length of hospice stay for patients with cancer was 52 days, while in Massachusetts the median length of stay across all medical conditions was 24 days.**^34^** In our study, patients enrolled earlier (only 11.4% enrolled 7 days prior; median hospice enrollment 27 days), which could reflect the slower disease pace of prostate cancer compared to other malignancies, or the biases of our academic clinical site and patient population. Importantly, we did not detect a change in contact days influenced by the type of therapies trialed before hospice, including clinical trials, although those cohorts were overall small. If anything, there was a trend toward decreased healthcare contact in the last quarter in patients who had tried three or more lines of metastatic treatment ahead of hospice enrollment, suggesting that heavily pre-treated patients were more comfortable with switching to hospice care. As previously mentioned, there have not been any studies analyzing healthcare contact around hospice, but existing literature has employed qualitative methods to describe patterns of end-of-life cancer care, emphasizing the need to have early informed discussions about patient preferences and the services hospice can offer to maximize time at home.**^32,35,36^**

This study had several limitations. Contact days were abstracted from available electronic medical record data limited to encounters within our healthcare system, which may underestimate the actual contact days if patients were seen in other healthcare facilities, such as rehabilitation facilities, nursing homes, or local hospitals. We also did not document the reason behind emergency room visits and hospitalizations, which could have been informative for patients and clinicians near the end-of-life. Moreover, we were unable to determine the level of care of patients after they enrolled in hospice. The assumption was that most patients spent their final days at home rather than in a hospice facility, but we had no access to that data. Finally, our results reflect contact days at a single academic institution with a limited number of clinicians and included patients who received treatment at a time with lower availability of effective lines of therapy in mCRPC than currently available. Thus, our study warrants replication in modern cohorts of patients with mCRPC at different care practices.

## Conclusion

Patients with mCRPC experience more contact days in their last 90 days of life compared to the beginning of their last year of life, driven by increased inpatient level of care. While treatment with ASI was associated with fewer contact days in the first quarter compared to infusional treatments, there were no differences in contact days between treatments in the last three months of life. Hospice enrollment was a protective factor in reducing healthcare contact, with earlier enrollment associated with greater reductions in contact days. These findings on expected time burden at end-stage mCRPC can provide early information to guide patient and clinician treatment decisions and end-of-life discussions.

## Data Availability

Data is available upon request with permission from the corresponding author.
